# Perception of diagnosis by family caregivers in severe brain injury patients in China

**DOI:** 10.1186/s12904-024-01482-8

**Published:** 2024-06-13

**Authors:** Yifan Yan, Meiqi Li, Jitka Annen, Wangshan Huang, Tiantian Cai, Xueying Wang, Xiaohua Hu, Steven Laureys, Haibo Di

**Affiliations:** 1 International Unresponsive Wakefulness Syndrome and Consciousness Science Institute, Hangzhou Normal University, Hangzhou, 310036 China; 2 School of Public Health, Zhejiang University, Hangzhou, China; 3 Department of Nursing, Hangzhou First People’s Hospital, Hangzhou, China; 4 Coma Science Group, GIGA-Consciousness, University of Liège, Liège, Belgium; 5 Centre du Cerveau2, University Hospital of Liège, Liège, Belgium; 6 School of Basic Medicine, Hangzhou Normal University, Hangzhou, China; 7 Department of Endocrinology, The First Affiliated Hospital of Chongqing Medical University, Chongqing, China; 8 Department of Rehabilitation, Hospital of Zhejiang People’s Armed Polic, Hangzhou, China; 9 CERVO Brain Research Centre, Laval University, Québec, Canada; 10 Department of radiology of Affiliated Hospital, Hangzhou Normal University, Hangzhou, China

**Keywords:** Severe brain injury, Diagnosis, Ethics, Family caregivers, Decision-making

## Abstract

**Objectives:**

Surrogate decision-making by family caregivers for patients with severe brain injury is influenced by the availability and understanding of relevant information and expectations for future rehabilitation. We aimed to compare the consistency of family caregivers’ perceptions with clinical diagnoses and to inform their expectation of prognosis in the future.

**Methods:**

The Coma Recovery Scale-Revised was used to assess the diagnosis of inpatients with severe brain injury between February 2019 and February 2020. A main family caregiver was included per patient. The family caregiver’s perception of the patient’s consciousness and expectations of future recovery were collected through questionnaires and compared consistently with the clinical diagnosis.

**Results:**

The final sample included 101 main family caregivers of patients (57 UWS, unresponsive wakefulness syndrome, 37 MCS, minimally conscious state, 7 EMCS, emergence from MCS) with severe brain injury. Only 57 family caregivers correctly assessed the level of consciousness as indicated by the CRS-R, showing weak consistency (Kappa = 0.217, *P* = 0.002). Family caregivers’ demographic characteristics and CRS-R diagnosis influenced the consistency between perception and clinical diagnosis. Family caregivers who provided hands-on care to patients showed higher levels of consistent perception (AOR = 12.24, 95% CI = 2.06-73.00, *P* = 0.006). Compared to UWS, the family caregivers of MCS patients were more likely to have a correct perception (OR = 7.68, 95% CI = 1.34–44.06). Family caregivers had positive expectations for patients’ recovery in terms of both communication and returning to normal life.

**Conclusion:**

Nearly half of family caregivers have inadequate understanding of their relative’s level of consciousness, and most of them report overly optimistic expectations that do not align with clinical diagnosis. Providing more medical information to family caregivers to support their surrogate decision-making process is essential.

**Supplementary Information:**

The online version contains supplementary material available at 10.1186/s12904-024-01482-8.

## Introduction

In China, more than 100,000 new cases of severe brain injury are reported annually, with patients typically distributed across various small and medium-sized hospitals [1]. Following severe brain injury, damage to neural pathways associated with arousal and awareness may result in disorders of consciousness (DoC), the mechanisms of which are unclear.

According to behavioral response, severe brain injury patients fall into several categories: coma, vegetative state (VS)/unresponsive wakefulness syndrome (UWS), minimally conscious state (MCS), and emergence from MCS (EMCS). Coma is clinically characterized by a complete loss of spontaneous or stimulus-induced arousal [2]. UWS is characterizing patients who are awake, but do not show any signs of awareness [3, 4]. Patients are categorized as being in a MCS when exhibit fluctuating non-reflexive behaviors in response to surroundings or stimuli [5]. If a patient progresses to the point of being able to use objects or communicate accurately, they are diagnosed in an EMCS and strictly not considered in a DoC any longer [6]. Despite the relatively clear classification, achieving an accurate diagnosis in clinical practice remains challenging. The diagnosis of patients with DoC is mainly based on their ability to follow commands. Medical professionals use various neurobehavioral scales to elicit the following behaviors: repeatable responses to visual, auditory, or noxious stimuli, object recognition and use, command following, (oro-)motor function and communication [7–13]. Evidence of one or more of these behaviors, as opposed to reflexes only, is considered indicative of consciousness and is used as a measure of consciousness assessment.

Since severe brain injuries usually occur unexpectedly, few patients have declared an advance directive to express treatment preferences, and therefore most medical decisions are made by surrogate decision-makers [14]. In China, surrogate decision-makers are usually family members of patients with DoC [15, 16]. Family members must have enough information about the diagnosis and prognosis to make medical decisions that are in the patient’s best interest. However, several studies revealed that family caregivers who were accompanying patients often had perceptions that were inconsistent with the patient’s diagnosis [17–19]. As reported by Ralf J and colleagues, in 76% of cases, relatives perceived the level of consciousness to be consistent with the diagnostic tests; in other cases, consciousness was mostly underestimated [19]. Underestimation of the patient’s level of consciousness will create pessimistic expectations for the family members and lead to premature withdrawal of life-sustaining treatment for their loved ones [20–22]. Conversely, if surrogates perceive that a patient can communicate better, they may believe their relatives will get better [23]. Therefore, overinterpreting the patient’s level of consciousness (e.g., communication ability) may lead to false hope, increasing the family caregiver’s fear of stagnation in rehabilitation and anxiety about future life [20–22]. Discrepancies between family members’ perceptions and diagnoses may result in serious doctor-patient conflicts and relate to patients’ medical decisions [24, 25]. Insight into the views of family caregivers is ethically crucial as it can facilitate communication between clinicians and caregivers, increasing the likelihood of reaching mutually agreeable decisions regarding the care of patients with severe brain injury.

We carried out a cross-sectional survey to assess first, the consistency between family caregivers’ perception and the patient’s level of consciousness based on a clinical diagnosis and second, the expectations of the family caregivers regarding the patient’s future recovery, especially regarding the recovery of communication and return to normal life.

## Methods

### Study design

We conducted an exploratory cross-sectional survey involving the primary family caregivers of severe brain injury patients. To confirm the patient’s level of consciousness, their behavioral responses were assessed using CRS-R by trained researchers. The CRS-R is a standardized neurobehavioral scale commonly used for assessing patients with DoC [26, 27]. It has demonstrated favorable sensitivity and specificity (Cronbach’s alpha = 0.84; Intraclass Correlation Coefficient = 0.87 in Chinese version), consisting of 23 items grouped into 6 scales that assess different domains: auditory, visual, motor, oromotor, communication, and arousal [26, 27]. Each patient underwent the CRS-R at least three times within a week, and the optimal diagnosis was defined as the patient’s final status. Patients with severe brain injury were classified into the following three categories based on the sub-items of the CRS-R according to the guidelines [27]: UWS, MCS, and EMCS. Moreover, we recorded the patients’ age, sex, and time since injuries.

The questionnaire for caregivers consisted of information below: firstly, family caregivers’ demographic information, including sex, age, religion, and education level; secondly, socioeconomic status, such as monthly income, relationship with patients, and type of occupation; thirdly, the family caregivers’ weekly care time, and care mode (e.g., hands-on care, where family caregivers directly care for patients; coordination care, where paid caregivers are involved in patient care).

To clarify the family’s perception of the patient’s state of consciousness, family members were asked to answer the question: “How do you perceive the patient’s current condition?”. Four closed answers were provided, reflecting specific characteristics of coma, UWS, MCS, and EMCS: (A) Without eyes open, in coma. (B) Opening eyes, but no sense of self or surroundings (corresponding to UWS). (C) Having significant response to surroundings or stimuli (corresponding to MCS). (D) Clear consciousness and accurate expression (corresponding to EMCS). To clarify the family’s expectations for future recovery, the family caregivers were asked to answer the question: “What do you think of the likelihood of the patient’s returning to communication/normal life in the future?”. Answers were presented using a numerical scale, with 1 indicating a tiny likelihood of recovery and 5 indicating a strong likelihood of future recovery. A score greater than the mean of 2.5 for the family primary caregiver’s expectation of future recovery was considered positive and less than 2.5 was considered negative. In addition, depressive symptom scores, anxiety symptom scores, and quality of life of the primary family caregivers were assessed using standardized scales to investigate mood burden. These were published in our previous study [28] (see supplementary 1: Questionnaire).

### Participants

Patients admitted to the rehabilitation hospital and their main family caregivers were included through a convenience sample during January 2019 and February 2020. Eligibility criteria included patients with severe brain injury diagnosed by a neurologist or clinician during inpatient rehabilitation in the acute (≤ 28 days by Chinese expert consensus) and chronic state (> 28 days) [29]. Patients who were medically unstable or experienced serious life-threatening complications were excluded from the study. Patient recruitment was conducted by the medical staff. Primary family caregivers include individuals who voluntarily and without compensation provide psychological, emotional, and practical assistance to their loved ones, typically being the patient’s relatives and friends [30]. Almost all patients have been transferred from the intensive care unit to the rehabilitation or neurology department, and their condition is relatively stable. Family members are allowed to enter the ward to provide adequate care for their loved ones. Family caregivers who participated in the study had to be fluent in Chinese and be able to read questions effortlessly. Only one main family caregiver was included per patient. In cases where multiple family caregivers were involved, the one providing the most care was included in the study.

### Ethical statement

Written informed consent to participate in the study was obtained from the primary family caregivers. This study was approved by the ethics committee of Hangzhou Normal University.

### Statistical analysis

Statistical analyses were performed using SPSS, Version 20.0 (SPSS Inc., Chicago, IL, USA). Descriptive methods were used for describing the demographic and basic information. The weighted kappa test was used to check consistency of the perception in family members and diagnosis by CRS-R. The Wilcoxon signed-rank test or Mann-Whitney U test was used to analyze the differences in family caregivers’ expectations for communication and normal life, as well as to examine potential factors influencing these expectations, e.g. sex and care mode. Kruskal-Wallis test was used to analyze the differences in expectation of recovery among diagnosis groups (UWS, MCS, and EMCS) as well as family caregiver’s demographic characteristics (e.g. educational level, relationship with patients, weekly care time, income, type of occupation and religion).

A generalized linear model (logistic regression) was used to predict the demographic characteristics that influence family members’ consistent perceptions (e.g., sex, age, educational level, relationship with patients, weekly care time, income, type of occupation, religion, care mode, patient’s age, and time brain injury). Binary logistic regression (using the enter method) was employed to predict the relationship between different clinical diagnoses and subscales of CRS-R (auditory, visual, motor, oromotor, communication and arousal) and the cognitive consistency of patients’ family caregivers. The study used a two-sided test, *P* < 0.05 was regarded as statistically significant.

## Results

One hundred and eight patients and their family members participated in the study. During the study, seven family members moved away. One hundred and one family members completed all the questions, with a response rate of 93.5%. Using the CRS-R to classify patients with severe brain injuries (age: 53 ± 15 years old; male: 74.3%, *n* = 75), they were categorized into UWS (56.4%, *n* = 57), MCS (36.6%, *n* = 37) and EMCS (6.9%, *n* = 7) (see supplementary 2: Basic information of severe brain injury patients). Nearly half (45.5%, *n* = 46) of the family caregivers were spouses of the patients (mean age: 48 ± 14). Family members provided care for the patients for at least 5 full days per week in the hospital, accounting for 71.3% of the caregivers (*n* = 72). The majority of the caregivers were freelancers (31.7%, *n* = 32), followed by retirees (22.8%, *n* = 23). Additionally, 58.4% of family caregivers (*n* = 59) had a monthly economic income of less than 1000 CNY (Table 1).

**Table 1:** Demographic information of family caregivers of patients (*N* = 101)

Demographics	*N* (%)
**Sex**	
Male	33 (32.7)
Female	68 (67.3)
**Age (years)** (𝑥̅ ± 𝑠) (Min – Max)	48.3 ± 13.9 (23–76)
**Religion**	
Non-religion	76 (75.2)
Have religion	25 (24.8)
Taoism	1 (1.0)
Buddhism	23 (22.8)
Christianity	1 (1.0)
**Education level**	
Primary school and below	34 (33.7)
Junior school	18 (17.8)
High school	31 (30.7)
Bachelor degree and upon	17 (17.8)
**Relationship with patients**	
Children	26 (25.7)
Spouse	46 (45.5)
Parents	18 (17.8)
Siblings	5 (5.0)
Others	6 (5.9)
Weekly care time*	
24 h and below	14 (13.9)
1–2 full days	10 (9.9)
3–4 full days	5 (5.0)
5 full days or more	72 (71.3)
**Income (CNY/month) (missing 3)**	
< 3000	59 (58.4)
3000–5000	26 (25.7)
5000–10,000	7 (6.9)
> 10,000	6 (5.9)
**Type of occupation**	
Full-time job	23 (22.8)
Part time job	4 (4.0)
Freelance	32 (31.7)
Student	1 (1.0)
Retired	23 (22.8)
Others	18 (17.8)
**Care mode**	
Hands-on care	77 (76.2)
Coordinated care	24 (23.8)

*Weekly care time refers to the amount of time primary family caregivers take care of patients each week

Regarding the perception of the patient’s diagnosis, 57 family members (56.4%) correctly assessed the level of consciousness as indicated by the CRS-R. On the contrary, 35 family members (34.7%) underestimated the patient’s current conscious state, and 7 family members (6.9%) overestimated the patient’s level of consciousness. Two participants did not select any category. In which, 8 family members (14.3%) believed that the patients who were in UWS were still in a coma; 22 MCS family members (61.1%) believed that their loved ones could not experience themselves and their surroundings. While there were 5 EMCS patients’ family members (71.4%) considered that their loved ones did not have clear consciousness or accurate expression (Table 2). Five UWS family members (8.9%) considered their loved ones had signs of consciousness. Two MCS family members (5.6%) thought that their loved ones had clear consciousness and could communicate accurately (Table 2). The consistency between the family’s perception of the patient’s consciousness and the patient’s diagnosis was weak (Kappa = 0.217, *P* = 0.002) (Table 2).

**Table 2:** The perception of family caregivers compared with the CRS-R results (*N* = 101)

Perception	Diagnosis			Kappa	*P* Value
	UWS (%)	MCS (%)	EMCS (%)		
Without eyes open, in coma	8 (14.3)	0 (0.0)	0 (0.0)	0.22	**0.002**
Opening eyes but can’t feel him/herself or surrounding	43 (76.8)	22 (61.1)	3 (42.9)		
Having significant response to surroundings and stimuli	4 (7.1)	12 (33.3)	2 (28.6)		
Clear consciousness and accurate expression	1 (1.8)	2 (5.6)	2 (28.6)		

The two participants did not select any category; No patient was diagnosed coma

CRS-R: Coma Recovery Scale-Revised; UWS: unresponsive wakefulness syndrome; MCS: minimally conscious state; EMCS: emergence from MCS.

Family caregivers’ demographic characteristics influence their accurate perception of the patient’s consciousness level (Table 3). Specifically, those who personally care for the patient are more likely to have a correct understanding of the patient’s consciousness level (AOR = 12.24, 95% CI = 2.06-73.00, *P* = 0.006). Family members who care for the patient 1–2 days per week (AOR = 13.10, 95% CI = 1.41-121.93, *P* = 0.024) or less than one day per week (AOR = 9.01, 95% CI = 1.12–72.80, *P* = 0.039) have a more accurate perception of the patient’s consciousness level compared to those who care for the patient more than five days per week. Freelancers (AOR = 6.80, 95% CI = 1.24–37.27, *P* = 0.027) also show a better ability to accurately perceive the patient’s consciousness level. Additionally, no other demographic factors such as age, education level, time since the patient’s injury, family income, religion, or the relationship between the family member and the patient were found to have a significant impact.

**Table 3:** Association of individual characteristics with perception consistency (*N* = 101)

Predict variables	β	SE	AOR (95% CI)	*P* Value
**Type of occupation**				
Full-time job	-0.06	0.93	0.95 (0.15–5.84)	0.953
Part-time job	-	-	-	1.000
Freelance	1.92	0.87	6.80 (1.24–37.27)	**0.027**
Student	-	-	-	1.000
Retired	0.71	0.86	2.04 (0.38–11.01)	0.409
Others	Ref			
**Care mode**				
Hands-on care	2.50	0.91	12.24 (2.06-73.00)	**0.006**
Coordinated care	Ref			
**Weekly care time**				
Less than a day	2.20	1.07	9.01 (1.12–72.80)	**0.039**
1–2 full days	2.57	1.14	13.10 (1.41-121.93)	**0.024**
3–4 full days	1.81	1.44	6.11(0.36-103.29)	0.210
5 full days or more	Ref			

β: Unstandardized Coefficient; SE: Standard Error; AOR: Adjusted Odd Ratio; CI: Confidence Interval. Among the significant variables, the reference groups are other occupations, coordinated care, and 5 full days or more

Adjusted for patient’s age, caregivers’ age, education level, time post-injury, religion, relationship with patients, and socioeconomic information such as income. Among all the predictor variables, type of occupation (goodness of fit: LR χ²=9.77, *P* = 0.082), mode of care (goodness of fit: LR χ²=9.59, *P* = 0.002), and weekly care time (goodness of fit: LR χ²=7.96, *P* = 0.047) were significant predictors of family caregivers’ cognitive consistency

Moreover, the CRS-R diagnosis and subscale scores also influence the accurate perception of the patient’s consciousness level by family caregivers (Table 4). Compared to UWS, the family caregivers of MCS patients were more likely to have a correct perception (OR = 7.68, 95% CI = 1.34–44.06, *P* = 0.022). The higher the scores on the visual (OR = 0.58, 95% CI = 0.43–0.79, *P* < 0.001) and arousal subscales (OR = 0.56, 95% CI = 0.32–0.99, *P* = 0.045) with patients, the greater the likelihood of erroneous perceptions among family members.

**Table 4:** The CRS-R diagnosis and subscale scores influence the accurate perception of the patient’s consciousness level by family caregivers (*N* = 101)

Predict variables	β	SE	OR (95% CI)	*P* Value
**Diagnosis with CRS-R**				
UWS	Ref			
MCS	2.04	0.89	7.68 (1.34–44.06)	**0.022**
EMCS	0.18	0.91	1.20 (0.20–7.10)	0.841
**Subscale scores with CRS-R**				
Auditory	-0.35	0.20	0.70 (0.48–1.04)	0.075
Visual	-0.54	0.16	0.58 (0.43–0.79)	**< 0.001**
Motor	-0.17	0.12	0.85 (0.67–1.07)	0.158
Oromotor	-0.46	0.33	0.63 (0.33–1.20)	0.160
Communication	-0.19	0.49	0.83 (0.32–2.17)	0.703
Arousal	-0.58	0.29	0.56 (0.32–0.99)	**0.045**

Binary logistic regression; Predictor group: Consistency

Among all the predictor variables, diagnosis (goodness of fit: χ²=19.78, *P* < 0.001), visual score (goodness of fit: χ²=13.78, *P* < 0.001), and arousal score (goodness of fit: χ²=4.37, *P* = 0.037) were significant predictors of family caregivers’ cognitive consistency

**Fig. 1:**
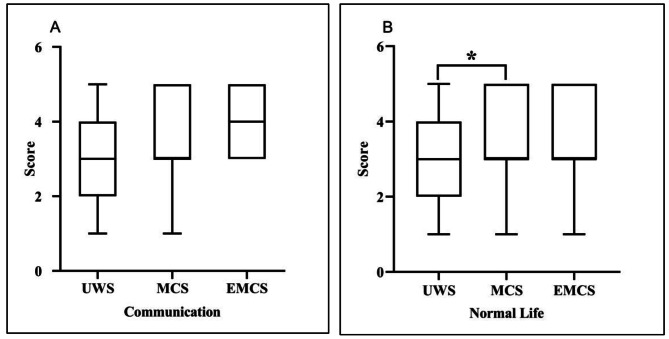
Family caregivers’ expectations for future recovery of patients (*N* = 101). *UWS family caregivers (mean score = 2.86, median score = 3, with interquartile range 2 to 4) had lower expectations than MCS group (mean score = 3.54, median score = 3, with interquartile range 3 to 5) in returning to normal life (H^2^=-2.587, *P =* 0.029)

Family caregivers had positive expectations for patients’ recovery, with median scores of 3 in communication and 3 in normal life, with an interquartile range of 3–4 and 2 to 4, respectively. They especially had positive expectations for the recovery of communication (Z=-2.165, *P* = 0.03). Despite differences in family caregivers’ expectations for patients with UWS, MCS, and EMCS regarding communication recovery (H^2^ = 6.950, *P* = 0.031), no statistically significant differences were found among the three groups after Bonferroni correction (all *P* > 0.05) (Fig. 1A). Based on the clinical assessment, UWS family caregivers (mean score = 2.86, median score = 3, with interquartile range 2 to 4) had lower expectations than MCS group (mean score = 3.54, median score = 3, with interquartile range 3 to 5) in returning to normal life (H^2^=-2.587, *P =* 0.029) **(**Fig. 1B**)**. Furthermore, family members’ perceptions influenced their expectations of communication recovery (H^2^ = 9.948, *P* = 0.019), with higher expectations among those who believed the individual had a clear response to surroundings or stimuli (mean score = 3.40, median score = 3, with interquartile range 3 to 4) than among those who believed the individual was in a coma (mean score = 2.75, median score = 2.5, with interquartile range 1.5 to 4.5) (H^2^=-2.879, *P* = 0.024). Compared to coordinated care (mean score = 2.88, median score = 3, with interquartile range 2 to 3), family members who were hands-on caregivers (mean score = 3.42, median score = 3, with interquartile range 3 to 5) for patients showed higher expectations of returning to previous quality of life (U = 664.000, *P* = 0.031), but there was no difference in expectations regarding the recovery of communication (U = 698.000, *P* = 0.055). There was no difference in family caregiver’s other demographic characteristics (see supplementary 3).

## Discussion

Healthcare surrogate decision-making for individuals with severe brain injury is influenced by a variety of factors, such as the availability and comprehensibility of relevant information and the expectations of future recovery [22]. This cross-sectional study focused on the perceptions of family caregivers regarding the diagnosis and prognosis of patients with severe brain injury. Our results revealed a weak concordance between the family’s perception of the patient’s state of consciousness and the clinical diagnosis. Notably, the family caregivers had high expectations for the patient’s future recovery, especially in terms of communication, and this expectation of recovery was influenced by factors such as clinical diagnosis and the perception of family caregivers. The results are highly beneficial for enhancing doctor-patient communication, reducing conflicts, and increasing the consistency of medical decision-making between both parties. Additionally, they hold significant implications for promoting palliative care for severe brain injury patients.

After various types of severe brain injury, most survivors rely on the care of family caregivers either in the hospital or at home. Our results showed that more than half of the family caregivers (56.4%) perceived the patient’s level of consciousness to be consistent with the diagnosis of the CRS-R, which is significantly lower than the concordance rate (76%) previously found in Germany [19]. The time post-injury in our patients was 141 ± 130 days, whereas in the study by Ralf J et al., patients were entered 6 months post-injury, long-term care by family caregivers may have gained more information about the illness to help them understand the conscious state of their loved one [19]. Additionally, there is no widely accepted uniform diagnostic criteria across different centers in China and significant variation in the professional levels of diagnosis assessment among clinicians [31]. This status quo may further hinder family caregivers’ understanding of the diagnosis.

On the other hand, there was a rather high variance between the family caregiver’s perception and the patient’s diagnosis. Specifically, we noted that 34.7% of family members underestimated the patient’s consciousness and 6.9% overestimated the patient’s consciousness. A possible explanation was that the family caregivers reported the initial diagnosis obtained from a medical professional and did not notice the patient’s slow change in consciousness over time [19]. In contrast to previous findings, we observed more family caregivers underestimating the patient’s ability rather than overestimating it [17]. Perhaps this difference can be explained methodologically: we asked family caregivers about their perceptions of the patient’s level of consciousness, which were based on four different diagnostic categories of coma, UWS, MCS, and EMCS, rather than simply asking whether the patients could establish communication with the environment, as Moretta et al. did [17]. Furthermore, the state of consciousness might have been underestimated by family members in the same way as the CRS-R, as it is inevitably influenced by inter-assessor variability, patients’ awakenings or consciousness fluctuations, movement defects, etc [18, 32, 33]. The overestimation reported by caregivers might be related to the notion that some patients show higher levels of consciousness with their loved ones. It has been shown that the presence of a caregiver positively influences the behavioral assessment of DoC patients: in 16% of the sample, the diagnosis changed from UWS to MCS, or from MCS to a severe disability when healthcare professionals assessed together with a caregiver [18]. We have similarly documented the contribution of family caregivers’ hands-on care for their loved ones to diagnosis and perception consistency. Given these results, we also support the idea of involving family caregivers in clinical assessment [34], as it may have potential benefits, such as satisfying the need for family members to be engaged in their relatives management and increasing the family’s perception of disease [35].

The results highlighted how clinical diagnoses of different patients with severe brain injury influence family caregivers’ perceptions of the patient’s current state. For example, the family caregivers of UWS patients were more likely to experience perceptions inconsistent with clinical diagnosis using CRS-R compared to MCS. This may be related to the residual abilities which patients present. Compared to UWS, MCS contains a broader classification: MCS+ (higher level of behavioral responses dependent on language function, like command following) and MCS- (lower level of behavioral responses independent from function, like visual tracking) [36, 37]. On the contrary, although patients with UWS cannot communicate with their surroundings or be aware of themselves, they still exhibit spontaneous reflex behaviors, such as yawning, grimacing, blinking, and so on, which may be mistakenly interpreted by family members as signs of consciousness. Similarly, this also explains why, in our study, an increase in scores on the visual and arousal subscales led to family members being more likely to develop cognitive dissonance with the clinical diagnosis. Additionally, in previous studies, the high misdiagnosis rate of UWS was also highlighted in neurology professionals [33, 38], which may have had a potential influence, as in most cases, family members’ information is based on medical professionals’ judgments. Once clinicians misdiagnose a patient’s level of consciousness and communicate this information to the family, the family members are likely to misinterpret the patient’s non-conscious signals as conscious ones (or vice versa). This can further exacerbate the discrepancy between the family’s perception and the patient’s actual condition. Therefore, the use of neuroimaging techniques such as fluorodeoxyglucose positron emission tomography (PDG-PET) and functional magnetic resonance imaging (fMRI) to assess brain function associated with consciousness or even establish communication with patients as a complementary means of detecting covert awareness remains necessary [39–41].

Similar to previous studies [19, 42], we also documented that the family caregivers always had positive expectations regarding the future prognosis of the patient. Most of the family caregivers who participated in our study were adult children (25.7%) and spouses (45.5%) of the patients, and the contribution of the variable of intimacy to high expectations has been demonstrated [23]. It was also explained that family caregivers have positive expectations for the future not only in terms of the patient’s recovery but also in terms of their own hopes for the future [23]. However, the prognosis for patients with severe brain injury is not always positive. Previous longitudinal studies have demonstrated that only 20% of patients in UWS achieved an improvement in consciousness between 14 and 28 months post-injury [43]. Although MCS patients may remain in the same state, they do have a better prognosis than UWS patients [44]. While maintaining hope may be necessary for family members to cope with adverse emotions in such a highly burdensome situation, the ineffectiveness of treatment may bring more suffering.

### Limitation

When interpreting our findings, several potential limitations need to be considered. We only report the opinions of family caregivers in specific regions regarding patients’ level of consciousness and future recovery expectations. We can not confirm whether the level of medical practice in different regions influenced these opinions. Although our results also showed that family caregivers of EMCS patients were more susceptible to comprehension inconsistency compared to the UWS, we remained cautious about the finding given the relatively small sample size (*n* = 7). A larger sample size is warranted to validate the results. Also, some patients only underwent the CRS-R assessment 3 times. However, recent studies have found that conducting the assessment 5 times in non-traumatic and 6 times in traumatic brain injury patients can significantly reduce misdiagnosis [32]. Therefore, behavioral diagnostic bias needs to be considered in the experimental design. We also lacked longitudinal data to further elucidate how family members’ perceptions evolved over time. Previous studies have reported that over time, family members’ medical knowledge increases, and unrealistic expectations decrease [23]. A further limitation of the present study arises from providing predetermined options for the perception of family caregivers. Leaving room for participants to add other considerations will therefore be a necessary step in future research.

## Conclusion

Nearly half of family caregivers have inadequate understanding of the patient’s level of consciousness and most of them report overly optimistic expectations that do not align with clinical diagnosis. The decision-making process used in surrogacy may suffer as a result. Family caregivers who are hands-on in caring for patients displayed more reliable clinical diagnostic judgment. We thus encourage family members to engage more in medical assessment to gain more information about medical care. Additionally, it is also essential that medical professionals ought to communicate with the patient’s family about the medical information of the patient in detail as soon as possible to avoid unrealistic expectations.

### Electronic supplementary material

Below is the link to the electronic supplementary material.

Supplementary Material 1

Supplementary Material 2

Supplementary Material 3

## Data Availability

The datasets used and/or analyzed during the current study are available from the corresponding author upon reasonable request.
