# Supplementary material for: Perception of diagnosis by family caregivers in severe brain injury patients in China
Source: BMC Palliat Care. 2024 Jun 13;23:148. doi: 10.1186/s12904-024-01482-8 (PMC11170822; doi:10.1186/s12904-024-01482-8)
Supplement: Supplementary file 1 — Supplementary Material 1 [file 12904_2024_1482_MOESM1_ESM.doc]

**Supplementary 1: Survey on the mental health of families of patients with disorders of consciousness**

1. Patient’s ID：______ Date：______

**Part 1：Demographic information**

1. **Age:** ______
2. **Sex:**  A. Male B. Female
3. **Relationship with patients:**
   1. Child
   2. Spouse
   3. Parents
   4. Siblings
   5. Friends
   6. Others
4. **Occupations:**
   1. Full-time job
   2. Part time job
   3. Freelance
   4. Students
   5. Retired
   6. Others
5. **Education level:**
   1. Primary school and below
   2. Junior school
   3. High school
   4. Bachelor degree
   5. Master’s degree and upon
   6. Others
6. **Religion：**
   1. [Taoism](javascript:;)
   2. Christianity
   3. Buddhism
   4. Others religion
   5. Non-religion
7. **Care mode:**
8. Hands-on care (take care patients by family caregivers)
9. Coordinated care (take care patients by paid caregivers, next item 9)
10. **Weekly care time：**
    1. 24 hours and below
    2. 1-2 full days
    3. 3-4 full days
    4. 5 full days or more
11. **Income (CNY/month):**
    1. <3000
    2. 3000-5000
    3. 5000-10000
    4. >10000

10. **How do you perceive the patient's current condition?**

A. Without eyes open, in coma

B. Opening eyes but can’t feel him/herself or surrounding

C. Having significant response to surroundings and stimuli

D. Clear consciousness and accurate expression

1. **How do you consider the patient's current mood state?**

A. Depression B. Anxiety C. None

1. **How do you think the patient's current condition is?**

A. Better condition than on admission

B. Same condition as on admission

C. Worse condition than on admission

1. **Please rate your patient's future condition:**
2. **What do you think of the likelihood of the patient’s returning to communication?**

□1 (tiny) □2 □3 □4 □5 (strong)

1. **What do you think of the likelihood of the patient’s returning to normal life in the future?**

□1 (tiny) □2 □3 □4 □5 (strong)

1. **Please rate the quality of your life in the last month?**

□0 (low) □1 □2 □3 □4 □5 □6 □7 □8 □9 □10（high）

**Part 2：Assessment mood state**

Directions: Answer the 9 questions and 7 questions below and tick the number that matches your choice.

| Over the last two weeks, how often have you been bothered by any of the following problems? | Score | | | |
| --- | --- | --- | --- | --- |
|  | Not at all | Several days | More than half the days | Nearly every day |
| 1. Little interest or pleasure in doing things | 0 | 1 | 2 | 3 |
| 2. Feeling down, depressed, or hopeless | 0 | 1 | 2 | 3 |
| 3. Trouble falling or staying asleep, or sleeping too much | 0 | 1 | 2 | 3 |
| 4. Feeling tired of having little energy | 0 | 1 | 2 | 3 |
| 5. Poor appetite or overeating | 0 | 1 | 2 | 3 |
| 6. Feeling bad about yourself -- or that you are a failure or having let yourself or your family down | 0 | 1 | 2 | 3 |
| 7. Trouble concentrating on things, such as reading the newspaper or watching television | 0 | 1 | 2 | 3 |
| 8. Moving or speaking so slowly that other people could have noticed. Or the opposite being so fidgety or restless that you have been moving a lot more than usual | 0 | 1 | 2 | 3 |
| 9. Thoughts that you would be better off dead, or of hurting yourself in some way | 0 | 1 | 2 | 3 |
| Over the last 2 weeks, how often have you been bothered by the following problems? | | | | |
| 1. Feeling nervous, anxious, or on edge | 0 | 1 | 2 | 3 |
| 2. Not being able to stop or control worrying | 0 | 1 | 2 | 3 |
| 3. Worrying too much about different things | 0 | 1 | 2 | 3 |
| 4. Trouble relaxing | 0 | 1 | 2 | 3 |
| 5. Being so restless that it's hard to sit still | 0 | 1 | 2 | 3 |
| 6. Becoming easily annoyed or irritable | 0 | 1 | 2 | 3 |
| 7. Feeling afraid as if something awful might happen | 0 | 1 | 2 | 3 |
