# Supplementary material for: Perception of diagnosis by family caregivers in severe brain injury patients in China
Source: BMC Palliat Care. 2024 Jun 13;23:148. doi: 10.1186/s12904-024-01482-8 (PMC11170822; doi:10.1186/s12904-024-01482-8)
Supplement: Supplementary file 2 — Supplementary Material 2 [file 12904_2024_1482_MOESM2_ESM.doc]

**Supplementary 2: Basic information of severe brain injury patients**

**Table 1** Disease information of patients (N=101)

| **Items** | **N (%)** |
| --- | --- |
| **Diagnosis** | |
| UWS | 57 (56.4) |
| MCS | 37 (36.6) |
| EMCS | 7 (6.9) |
| **Sex** | |
| Male | 75 (74.3) |
| Female | 26 (25.7) |
| **Age (years)** | 𝑥̅ ± 𝑠 (Min – Max) |
|  | 52.5±14.5 (16 – 80) |
| **Etiology** |  |
| Trauma | 49 (48.5) |
| Anoxia | 4 (4.0) |
| Stroke | 43 (42.6) |
| Other | 5 (5.0) |
| **Time since injury* (days)** | 𝑥̅ ± 𝑠 (Min - Max) |
|  | 141.4±130.6 (19-821) |

* Time since injury means the time between the brain injury and family caregivers filling the questionnaires

UWS: unresponsive wakefulness syndrome

MCS: minimally conscious state

EMCS: emerging from MCS

**Table 2** Detailed information of severe brain injury patients

| No. | Etiology | Age | Time post-injury^*^ | Sex | Diagnosis | CRS-R score | | | | | |
| --- | --- | --- | --- | --- | --- | --- | --- | --- | --- | --- | --- |
|  |  |  |  |  |  | [Auditory](javascript:;) | Visual | Motor | Oromotor | Communication | Arousal |
| 1 | Trauma | 44 | 67 | M | UWS | 1 | 1 | 0 | 1 | 0 | 2 |
| 2 | Trauma | 53 | 31 | F | MCS- | 2 | 2 | 4 | 1 | 0 | 2 |
| 3 | Trauma | 67 | 183 | M | UWS | 0 | 0 | 2 | 0 | 0 | 0 |
| 4 | Other | 39 | 141 | M | EMCS | 3 | 5 | 6 | 3 | 2 | 3 |
| 5 | Anoxia | 30 | 58 | M | UWS | 0 | 1 | 1 | 1 | 0 | 0 |
| 6 | Other | 63 | 210 | M | UWS | 0 | 0 | 2 | 0 | 0 | 2 |
| 7 | Stroke | 69 | 88 | M | UWS | 1 | 1 | 0 | 1 | 0 | 2 |
| 8 | Other | 45 | 177 | M | MCS- | 1 | 0 | 5 | 1 | 0 | 2 |
| 9 | Stroke | 68 | 26 | M | UWS | 0 | 1 | 2 | 0 | 0 | 0 |
| 10 | Stroke | 79 | 296 | M | UWS | 0 | 0 | 2 | 1 | 0 | 0 |
| 11 | Trauma | 47 | 21 | F | UWS | 0 | 0 | 2 | 0 | 0 | 0 |
| 12 | Cerebral hernia | 70 | 296 | M | UWS | 0 | 1 | 1 | 1 | 0 | 2 |
| 13 | Trauma | 48 | 157 | M | UWS | 1 | 1 | 2 | 0 | 0 | 1 |
| 14 | Stroke | 64 | 114 | F | UWS | 0 | 0 | 0 | 1 | 0 | 2 |
| 15 | Stroke | 56 | 127 | M | UWS | 0 | 1 | 0 | 0 | 0 | 2 |
| 16 | Stroke | 40 | 27 | M | EMCS | 4 | 1 | 6 | 1 | 0 | 3 |
| 17 | Trauma | 62 | 222 | F | UWS | 1 | 0 | 2 | 0 | 0 | 1 |
| 18 | Trauma | 74 | 158 | M | UWS | 1 | 1 | 2 | 0 | 0 | 2 |
| 19 | Trauma | 54 | 230 | M | UWS | 0 | 0 | 2 | 1 | 0 | 2 |
| 20 | Stroke | 54 | 44 | M | MCS- | 2 | 3 | 3 | 0 | 0 | 3 |
| 21 | Brain tumor | 32 | 318 | M | MCS- | 1 | 3 | 0 | 1 | 0 | 2 |
| 22 | Other | 34 | 94 | M | UWS | 0 | 0 | 2 | 1 | 0 | 1 |
| 23 | Trauma | 36 | 105 | M | MCS- | 2 | 3 | 5 | 1 | 0 | 2 |
| 24 | Trauma | 48 | 100 | M | UWS | 1 | 1 | 1 | 1 | 0 | 1 |
| 25 | Stroke | 52 | 32 | F | MCS- | 0 | 1 | 5 | 1 | 0 | 2 |
| 26 | Stroke | 65 | 47 | F | MCS- | 2 | 3 | 2 | 1 | 0 | 2 |
| 27 | Stroke | 59 | 80 | M | MCS+ | 3 | 4 | 5 | 2 | 0 | 3 |
| 28 | Trauma | 43 | 112 | M | UWS | 0 | 0 | 0 | 1 | 0 | 1 |
| 29 | Stroke | 54 | 77 | M | MCS+ | 3 | 4 | 2 | 1 | 0 | 2 |
| 30 | Stroke | 56 | 74 | M | UWS | 0 | 1 | 0 | 2 | 0 | 2 |
| 31 | Trauma | 71 | 115 | F | UWS | 0 | 1 | 1 | 1 | 0 | 2 |
| 32 | Anoxia | 61 | 40 | M | UWS | 1 | 0 | 0 | 1 | 0 | 1 |
| 33 | Trauma | 37 | 109 | M | MCS- | 1 | 3 | 1 | 1 | 0 | 1 |
| 34 | Trauma | 45 | 115 | M | UWS | 0 | 0 | 0 | 2 | 0 | 1 |
| 35 | Trauma | 51 | 38 | M | UWS | 0 | 0 | 2 | 0 | 1 | 2 |
| 36 | Brain tumor | 72 | 446 | M | MCS- | 2 | 3 | 0 | 1 | 0 | 2 |
| 37 | Stroke | 49 | 54 | M | UWS | 0 | 0 | 0 | 1 | 0 | 0 |
| 38 | Stroke | 43 | 347 | M | MCS- | 1 | 3 | 2 | 0 | 0 | 2 |
| 39 | Brain tumor | 66 | 152 | M | MCS+ | 2 | 3 | 5 | 1 | 0 | 2 |
| 40 | Trauma | 67 | 73 | M | UWS | 1 | 1 | 1 | 1 | 0 | 0 |
| 41 | Stroke | 63 | 20 | F | UWS | 0 | 1 | 1 | 1 | 0 | 2 |
| 42 | Trauma | 64 | 195 | M | UWS | 1 | 1 | 2 | 1 | 0 | 2 |
| 43 | Trauma | 33 | 53 | M | EMCS | 2 | 4 | 6 | 1 | 1 | 2 |
| 44 | Stroke | 58 | 282 | M | UWS | 1 | 1 | 2 | 0 | 0 | 2 |
| 45 | Trauma | 70 | 55 | M | MCS- | 3 | 3 | 1 | 1 | 0 | 1 |
| 46 | Trauma | 64 | 89 | M | MCS- | 2 | 1 | 5 | 1 | 0 | 2 |
| 47 | Stroke | 26 | 36 | F | UWS | 0 | 0 | 2 | 0 | 0 | 1 |
| 48 | Trauma | 65 | 56 | F | UWS | 0 | 0 | 2 | 0 | 0 | 2 |
| 49 | Stroke | 50 | 296 | M | UWS | 0 | 0 | 0 | 0 | 0 | 2 |
| 50 | Stroke | 61 | 45 | M | MCS- | 1 | 3 | 0 | 1 | 0 | 1 |
| 51 | Stroke | 75 | 107 | M | MCS- | 1 | 3 | 1 | 0 | 0 | 2 |
| 52 | Stroke | 56 | 351 | M | MCS+ | 3 | 3 | 0 | 1 | 1 | 2 |
| 53 | Trauma | 24 | 680 | M | MCS- | 2 | 3 | 0 | 1 | 0 | 2 |
| 54 | Trauma | 23 | 821 | M | UWS | 0 | 0 | 2 | 0 | 0 | 2 |
| 55 | Trauma | 77 | 192 | M | MCS- | 0 | 3 | 0 | 1 | 0 | 2 |
| 56 | Stroke | 53 | 117 | F | UWS | 0 | 1 | 1 | 1 | 0 | 2 |
| 57 | Trauma | 55 | 156 | F | UWS | 1 | 1 | 2 | 1 | 0 | 1 |
| 58 | Stroke | 56 | 25 | F | UWS | 0 | 0 | 1 | 0 | 0 | 1 |
| 59 | Trauma | 37 | 89 | M | UWS | 0 | 1 | 2 | 1 | 0 | 2 |
| 60 | Stroke | 48 | 56 | M | EMCS | 3 | 5 | 6 | 2 | 1 | 2 |
| 61 | Stroke | 44 | 424 | M | UWS | 0 | 1 | 2 | 0 | 0 | 2 |
| 62 | Trauma | 55 | 19 | M | EMCS | 3 | 0 | 6 | 3 | 2 | 4 |
| 63 | Stroke | 62 | 106 | M | MCS- | 0 | 3 | 2 | 1 | 0 | 1 |
| 64 | Trauma | 59 | 79 | M | UWS | 0 | 0 | 2 | 1 | 0 | 2 |
| 65 | Anoxia | 68 | 133 | M | UWS | 1 | 1 | 2 | 1 | 0 | 2 |
| 66 | Other | 36 | 102 | M | UWS | 0 | 0 | 0 | 0 | 0 | 2 |
| 67 | Stroke | 57 | 58 | M | MCS- | 2 | 3 | 2 | 1 | 0 | 2 |
| 68 | Stroke | 48 | 43 | M | EMCS | 1 | 4 | 6 | 1 | 0 | 3 |
| 69 | Trauma | 62 | 25 | M | MCS+ | 2 | 1 | 5 | 1 | 1 | 2 |
| 70 | Stroke | 32 | 206 | M | UWS | 0 | 0 | 2 | 0 | 0 | 2 |
| 71 | Trauma | 67 | 32 | M | MCS- | 1 | 3 | 2 | 1 | 0 | 1 |
| 72 | Trauma | 57 | 134 | F | MCS- | 2 | 3 | 2 | 1 | 0 | 2 |
| 73 | Trauma | 17 | 201 | F | MCS- | 2 | 4 | 2 | 1 | 0 | 2 |
| 74 | Trauma | 71 | 89 | F | UWS | 1 | 0 | 2 | 1 | 0 | 2 |
| 75 | Encephaledema | 55 | 253 | F | UWS | 1 | 1 | 2 | 1 | 0 | 1 |
| 76 | Stroke | 72 | 70 | M | UWS | 0 | 0 | 1 | 0 | 0 | 2 |
| 77 | Other | 44 | 126 | M | UWS | 0 | 0 | 0 | 1 | 0 | 1 |
| 78 | Stroke | 48 | 178 | F | MCS- | 0 | 3 | 0 | 1 | 0 | 2 |
| 79 | Trauma | 59 | 60 | F | UWS | 1 | 1 | 1 | 1 | 0 | 2 |
| 80 | Stroke | 49 | 144 | F | MCS- | 2 | 3 | 0 | 1 | 0 | 2 |
| 81 | Trauma | 38 | 138 | M | UWS | 2 | 1 | 2 | 1 | 0 | 1 |
| 82 | Stroke | 52 | 152 | M | MCS+ | 1 | 1 | 1 | 1 | 1 | 2 |
| 83 | Stroke | 42 | 218 | M | UWS | 0 | 0 | 2 | 1 | 0 | 1 |
| 84 | Trauma | 58 | 134 | M | MCS- | 2 | 3 | 2 | 1 | 0 | 2 |
| 85 | Trauma | 64 | 242 | F | UWS | 1 | 1 | 1 | 0 | 0 | 1 |
| 86 | Trauma | 69 | 91 | M | MCS- | 2 | 3 | 2 | 1 | 0 | 2 |
| 87 | Stroke | 38 | 56 | M | UWS | 0 | 0 | 0 | 0 | 0 | 1 |
| 88 | Stroke | 80 | 58 | F | UWS | 0 | 0 | 1 | 1 | 0 | 0 |
| 89 | Stroke | 57 | 27 | M | EMCS | 4 | 5 | 6 | 3 | 2 | 3 |
| 90 | Stroke | 61 | 419 | M | UWS | 0 | 0 | 0 | 0 | 0 | 1 |
| 91 | Stroke | 46 | 74 | M | MCS+ | 3 | 3 | 1 | 1 | 0 | 2 |
| 92 | Trauma | 66 | 56 | M | MCS- | 1 | 4 | 4 | 1 | 0 | 3 |
| 93 | Trauma | 47 | 214 | F | UWS | 0 | 1 | 1 | 1 | 0 | 1 |
| 94 | Trauma | 41 | 59 | M | MCS- | 1 | 1 | 3 | 1 | 0 | 2 |
| 95 | Trauma | 16 | 41 | F | UWS | 0 | 1 | 1 | 1 | 0 | 1 |
| 96 | Trauma | 16 | 154 | F | UWS | 2 | 1 | 1 | 2 | 0 | 2 |
| 97 | Anoxia | 59 | 145 | M | MCS- | 2 | 3 | 3 | 0 | 0 | 2 |
| 98 | Trauma | 56 | 35 | M | UWS | 0 | 0 | 1 | 1 | 0 | 1 |
| 99 | Trauma | 32 | 211 | F | UWS | 1 | 1 | 2 | 1 | 0 | 1 |
| 100 | Stroke | 32 | 62 | M | MCS- | 1 | 3 | 0 | 1 | 0 | 2 |
| 101 | Trauma | 46 | 293 | M | MCS- | 1 | 3 | 1 | 1 | 0 | 1 |
