# Supplementary material for: Perception of diagnosis by family caregivers in severe brain injury patients in China
Source: BMC Palliat Care. 2024 Jun 13;23:148. doi: 10.1186/s12904-024-01482-8 (PMC11170822; doi:10.1186/s12904-024-01482-8)
Supplement: Supplementary file 3 — Supplementary Material 3 [file 12904_2024_1482_MOESM3_ESM.docx]

|  | Communication | | | Normal life | | |
| --- | --- | --- | --- | --- | --- | --- |
| Items | Rank | H/U | *P* Value | Rank | H/U | *P* Value |
| Sex |  |  |  |  |  |  |
| Male | 47.74 | 1014.500 | 0.408 | 48.48 | 1039.000 | 0.532 |
| Female | 52.58 |  |  | 52.22 |  |  |
| Religion |  |  |  |  |  |  |
| Non-religion | 50.13 | 0.288 | 0.591 | 48.23 | 2.828 | 0.093 |
| Have religion | 53.50 |  |  | 59.00 |  |  |
| Education level |  |  |  |  |  |  |
| Primary school and below | 47.16 | 4.646 | 0.326 | 46.56 | 3.189 | 0.527 |
| Junior school | 46.25 |  |  | 47.89 |  |  |
| High school | 51.00 |  |  | 53.82 |  |  |
| Bachelor degree | 62.56 |  |  | 59.75 |  |  |
| Master degree and above | 66.50 |  |  | 40.75 |  |  |
| Relationship |  |  |  |  |  |  |
| Children | 45.40 | 7.646 | 0.105 | 45.19 | 8.945 | 8.945 |
| Spouse | 47.03 |  |  | 47.57 |  |  |
| Parents | 59.42 |  |  | 54.58 |  |  |
| Siblings | 71.20 |  |  | 73.80 |  |  |
| Others | 63.58 |  |  | 72.75 |  |  |
| Income |  |  |  |  |  |  |
| <3000 | 48.03 | 0.826 | 0.662 | 47.91 | 0.544 | 0.762 |
| 3000-5000 | 49.85 |  |  | 52.54 |  |  |
| 5000-10000 | 55.46 |  |  | 50.65 |  |  |
| Weekly care time |  |  |  |  |  |  |
| 24 hours and below | 56.54 | 3.067 | 0.381 | 56.29 | 2.994 | 0.393 |
| 1-2 full days | 37.40 |  |  | 37.25 |  |  |
| 3-4 full days | 52.70 |  |  | 54.50 |  |  |
| 5 full days or more | 51.69 |  |  | 51.64 |  |  |

**Supplementary 3:** Potential factors of family caregivers’ demographics in expectations for patients’ recovery
